# Supplementary figures and images for: Synthetic amyloid beta does not induce a robust transcriptional response in innate immune cell culture systems
Source: J Neuroinflammation. 2022 Apr 23;19:99. doi: 10.1186/s12974-022-02459-1 (PMC9034485; doi:10.1186/s12974-022-02459-1)

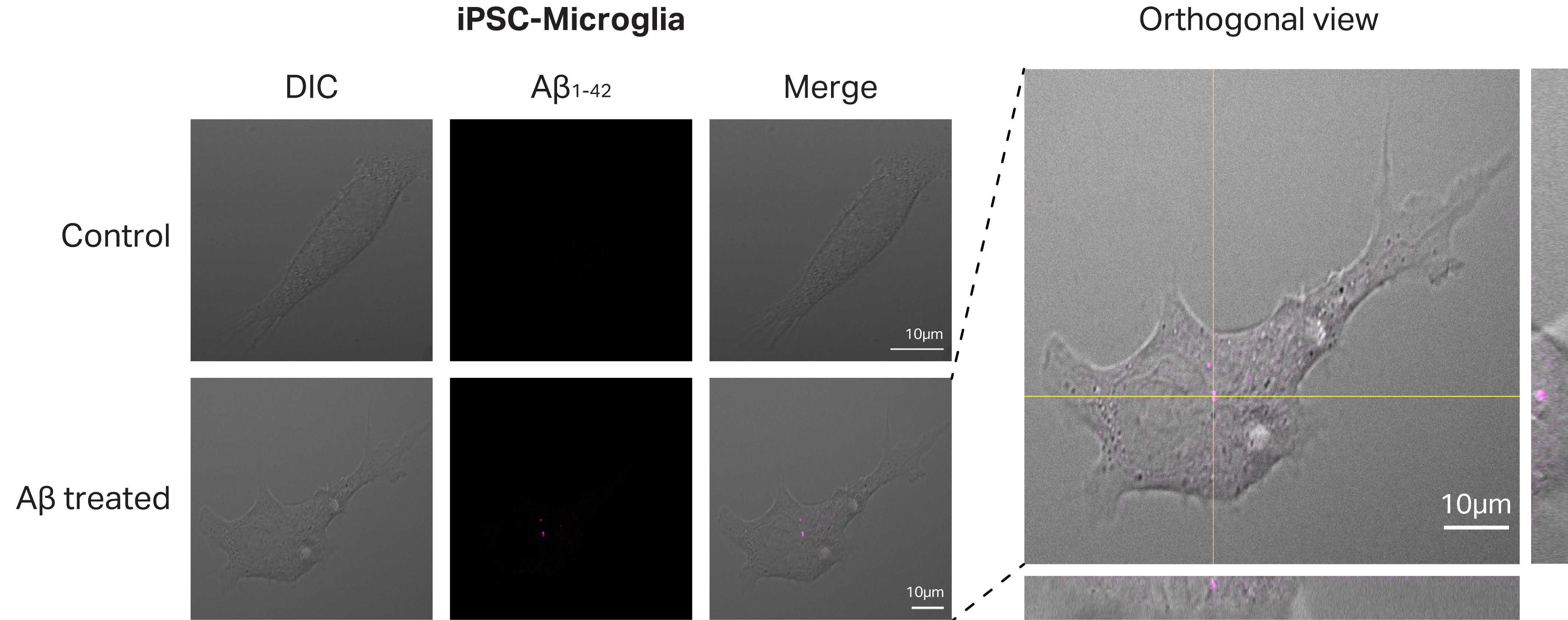

Supplement: Supplementary file 1 — Additional file 1: Figure S1. Aβ is incorporated into IMGLs. IMGLs incubated with 2 µg/mL of oligomeric β-Amyloid1-42-HiLyte Fluor 555 for 1 h. Confocal images were acquired on an inverted Zeiss 800 laser scanning confocal microscope. Scale bar, 10 µm. [file 12974_2022_2459_MOESM1_ESM.png]

# THP-1 macrophages + 24 hour LPS/IFN $\gamma$

RNA-Seq  
qPCR

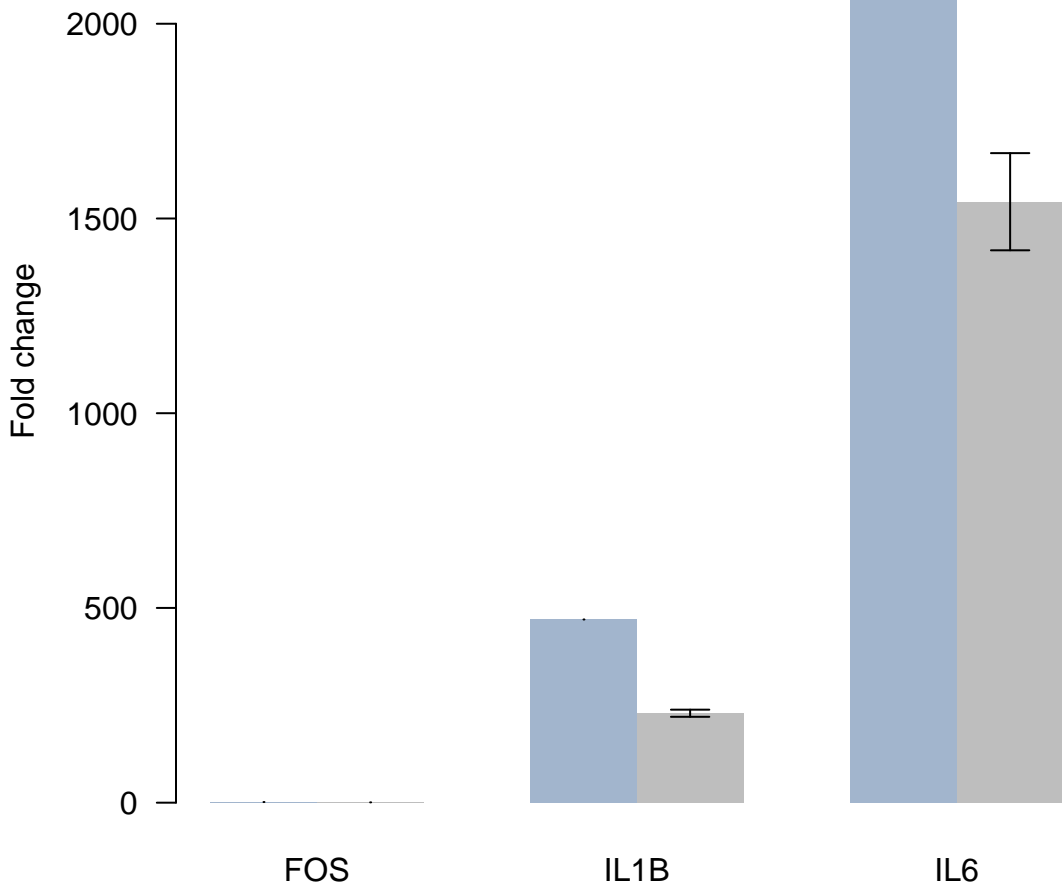

Supplement: Supplementary file 2 — Additional file 2: Figure S2. qPCR for selected genes confirms RNA-seq results. THP-1 macrophages were treated with 10 ng/ml LPS + 20 ng/ml INFγ for 24 h. The fold-changes of 3 genes were measured with qPCR in duplicate and compared to fold-changes detected by RNA-seq. [file 12974_2022_2459_MOESM2_ESM.pdf]
